# Extended fed-batch fermentation of a C5/C6 optimised yeast strain on wheat straw hydrolysate using an online refractive index sensor to measure the relative fermentation rate

**DOI:** 10.1038/s41598-020-63626-z

**Published:** 2020-04-21

**Authors:** Jan Dines Knudsen, Birgitte Rønnow

**Affiliations:** Terranol A/S, A.C. Meyers Vænge 15, 2450 Copenhagen, Denmark

**Keywords:** Biotechnology, Applied microbiology, Biofuels, Bioalcohols

## Abstract

In the production of 2^nd^ generation ethanol, using *Saccharomyces cerevisiae*, the highest productivity obtained using C5/C6 fermenting yeast is in the co-fermentation phase, in which xylose and glucose are fermented simultaneously. Extending this phase in a fed-batch process increases the yield, rate and additionally reduces needed yeast amount for pitching. Extending this phase, as long as possible, would further enhance yield and economy of the process. To realise the concept a fermentation monitoring technique was developed and applied. Based on online measured refractive index an optimal residual sugar concentration could be maintained in the primary fermentor during the feed phase, requiring little knowledge of the nature of the substrate. The system was able to run stably for at least five fermentor volumes giving an ethanol yield >90% throughout the run. This was achieved with addition of only urea to the wheat straw hydrolysate and with an initial yeast pitch of 0.2 g/L total of finished broth. It has the potential to improve the fermentation technology used in fuel ethanol plants, which could help to meet the growing demand for more sustainable fuels.

## Introduction

There is a growing need for sustainable liquid biofuels to be used in the transportation sector to replace fossil fuels such as 2^nd^ generation ethanol (2G ethanol) bioethanol, which is produced from renewable feedstock^1^. A promising feedstock for 2G ethanol is biomass from agricultural or forestry residues^2^. Until recently, lignocellulosic 2G ethanol production has not been economically feasible due to low profitability – for thorough reviews see^3,4^. Various approaches attempted in order to increase the profitability, have mainly focused on development of effective pre-treatment methods to facilitate hydrolysis and fermentation^5^, reduction of enzyme costs and loading^6^, modification and improvement of strains that are efficient in co-fermentation of pentose and hexose sugars under inhibitory conditions^7–10^ or media formulation^11^. Research has also been directed towards processing configurations, which are mainly focused on the relationship between hydrolysis and batch fermentation, such as separate hydrolysis and fermentation^12^, hybrid hydrolysis and fermentation^13^, simultaneous saccharification and fermentation^14^, and consolidated bioprocessing^15^. However, not much effort has been spent on the development of bioprocessing strategies, which increase productivity through fermentation methodologies. Batch operation is widely used for ethanol fermentation due to its simplicity. However, ethanol yields are typically lower than what can be obtained employing fed-batch and continuous processes^16^. Furthermore, downtime is necessary after each fermentation to clean up the reactor and prepare for the next fermentation^17^. Thus, one approach to improve productivity of batch fermentation would be to reduce fermentation time and downtime and extend the most productive phase of the fermentation. In addition, to achieve the desired levels of ethanol production, industrial ethanol facilities employ multiple batch bioreactors to ensure an uninterrupted flow of fermentation product for distillation. Correspondingly, microbial propagation^18^, a lengthy and multi-stage scale-up process that provides fermentors with seed culture, is needed for every batch fermentation cycle. Could this requirement be omitted, the costs would be lowered significantly: Published data regarding operating expenditures (OPEX) for full scale 2G ethanol production, in terms of enzyme and yeast purchase, are scarce. However, based on the few public records 2G ethanol production requires 10 ₵ of enzymes and 6 ₵ of yeast per litre of produced ethanol, thus far exceeding the analogous OPEX needed for running a 1^st^ generation (1G) grain-based plant^19^. It should, however, be kept in mind that breaking down cellulose is biochemically very different than hydrolysing starch, and one should be careful about making a one to one comparison in terms of enzyme loads. One approach, to lower the production costs, is to use a fed-batch fermentation instead. In a fed-batch fermentation, there are two phases in addition to the batch i.e. the feed and end phase. The three phases are characterised by different ethanol yields and productivities^20^. Thus, expanding the most productive fermentation phase is of vital interest, and not only is there a need to extend this phase, but also to develop a feeding strategy that ensures the highest possible ethanol productivity throughout the feed phase of the fermentation. However, implementing a full continuous process requires great demands on sterilization and contamination control. But there is a window of opportunity for an enhanced fermentation process by extending the fed-batch mode into a continuous mode for a limited time. This requires an on-line method that allows for continuous insight into the substrate and product concentrations of the fermentation: It is already known that the unitless value of refractive index (*nD*) of an aqueous solution is directly correlated to the sugar concentration by its Brix value^21^ and it is used in the brewing industry for monitoring the fermentations^22^. By measuring the refractive index value, the concentrations of solutions can be estimated and, more importantly, fermentation transitions can be detected. The present work proposes a new fermentation concept as a solution to the outlined challenges encountered by the 2G ethanol industry. The concept is referred to as “CoRyFee”^23^. It utilizes a continuous fermentation regime where a primary fermentation tank is filled, at such a rate that the optimal ethanol productivity is maintained and emptied into two parallel connected holding tanks. Filling of the holding tanks is alternating where one is filled while the second is emptied to the distillation unit (and vice versa). This is made possible by the development of a new feeding strategy, which is based on real-time measurements of the sugar concentration in the culture. This is, to the best of the authors knowledge, the first time that the refractive index has been applied to monitoring and guiding a fuel ethanol fermentation. As microbial host the C5/C6 fermenting cV-110 *Saccharomyces cerevisiae* strain was used. It is a strain developed and proprietary to Terranol A/S^9,10^. It is based on a *S. cerevisiae* Meyen ex E.C. Hansen strain. The organism has been genetically modified and in addition, has undergone extensive evolutionary engineering in order to optimise its C5/C6 co-fermentation capacity, the tolerance towards inhibitors and eliminating unwanted side products.

## Results

### Identification of a scalable online fermentation monitoring system

To establish a feeding system that would enable a constant sugar concentration in the fermentor, at which the fermentation rate is at its highest i.e. optimum, the need for an online monitoring system was addressed. The system should be scalable so that it could easily take the leap from laboratory to industrial scale. Several monitoring systems were evaluated e.g. measurement of gas flow and CO_2_ emission from the reactor, both of which can be used to monitor the rate of fermentation (data not shown). The systems were, however, regarded as suboptimal as difficulties were foreseen, and they did not have the potential of scaling up. Instead attention was turned to measurement of the unitless value of refractive index – referred to as *nD*. A standard curve of sugar concentration (g/L) in water vs. the solutions *nD*-value, at temperature *T* i.e. *nD(T)* and the following standard curve was obtained:

$${\rm{RI}}({\rm{g}}/{\rm{L}})=7068.9\times nD(T)-9423.5$$

This equation directly correlates the sugar concentration in water at temperature *T –* i.e. *nD(T)* to an RI value. In an aqueous sugar solution, the RI value corresponds directly to the total sugar concentration i.e. 1 g/L of glucose equals 1 RI (g/L) value. Same concentrations of glucose and xylose give the same RI value e.g. 1 g/L of glucose equals 1 RI (g/L) value equals 1 g/L xylose. Other compounds in aqueous solution such as salts and acids etc., referred to as background noise, also affect the RI value if present, but particulate matters, including formed yeast, will not. However, products formed during the bioconversion e.g. glycerol and ethanol will affect the RI value e.g. 1 g/L glucose gives the same RI signal as 2 g/L ethanol or 1.1 g/L glycerol. This imposes limitations in terms of interpreting the calculated RI as an exact measure of the sugar concentration during a fermentation on complex media. However, provided that the concentrations of sugar, ethanol, and glycerol are stable the RI value will remain unchanged.

### Verification of the RI monitoring system

**Figure 1:**
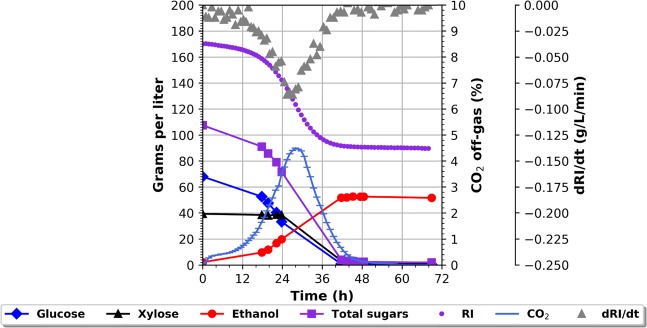
Batch fermentation. The fermentation conditions were kept at 32 °C, pH 5.5. Terranol’s cV-110 yeast strain was used with a total pitch of 1 g/L i.e. 1 g/L initially in the batch. The initial batch was composed of 1 L wheat straw hydrolysate (71 g/L glucose, 40 g/L xylose, 6.5 g/L acetic acid) supplemented with urea at a concentration of 3 g/L.

As verification of the RI monitoring system a batch fermentation was performed. The fermentation conditions were kept at 32 °C, pH 5.5. The cV-110 yeast strain was used with a total pitch of 1 g/L i.e. 1 g/L initially in the batch. The initial batch was composed of 1 L wheat straw hydrolysate, containing both C6 and C5 sugars, supplemented with urea at a concentration of 3 g/L. An external refractometer was connected to the fermentor, by a sterile tube, so that the broth could continuously be pumped out of the fermentor and passed by the measuring cell of the refractometer before being pumped back into the reactor. The measuring cell would measure the refractive index, *nD*, and the temperature, *T*, of the broth. The online refractometer was connected to the supervisory control and data acquisition system (BioPAT, MFCS/win) via an open platform communication server. It allowed for monitoring the *nD(T)* value and hence the calculation of RI. Additionally, CO_2_ and ethanol in the off gas were analysed online and high-pressure liquid chromatography (HPLC) sampling for sugars and metabolites were conducted at regular time intervals. The CO_2_ and HPLC measurements are established methods for measuring relative fermentation rate and metabolite concentrations in the broth, respectively. The results are shown in Fig. 1. The initial RI value is close to 170 g/L even though the sugar concentration (glucose + xylose) is only 110 g/L. The 60 g/L sugar equivalents difference between the measured RI sugar units and actual sugar concentration is due to the before mentioned background noise – salts etc. However, this background is constant for the specific substrate, and can, if desired by the user, be compensated for in future measurements. Also, the background noise does not affect the change in sugar concentration. As the fermentation progresses the RI value follows an S-shaped curve, which closely resembles the curve of the total sugar concentration also seen in the figure. As the sugars are depleted the RI curve reaches a steady state around 90 g/L. The reason why it does not reach 60 g/L, i.e. the before mentioned background, is that the main fermentation products, ethanol and glycerol, also add to the *nD* reading and hence to the RI value as previously described. It is worth noticing that the cV-110 strain can consume all glucose and xylose and has no detectable xylitol production. The CO_2_ concentration of the exhaust gas indicates the relative sugar conversion rate, the higher the concentration of CO_2_ the higher the conversion rate. The same rate is indicated by the RI and dRI/dt curves; the steeper the curve the faster the sugar conversion and hence the total ethanol production rate. The peak of the CO_2_ curve coincides with the minimum value of the dRI/dt curve. The RI reading thus gives real-time insight into the sugar conversion rate of the fermentation, albeit a relative one.

### Identifying the timing of highest productivity in a batch fermentation

Previously the CO_2_ emission gas has successfully been applied for the determination, and maintaining, of the optimal sugar concentration that will enable the fastest co-fermentation rate^24^. In short, the CO_2_ emission peak indicates the time of the highest overall sugar conversion. However, this concept has some drawbacks; (1) There is a certain lag-phase on the CO_2_ signal as the sugar needs to be metabolised, the CO_2_ produced and transported to the detector. (2) The CO_2_ is a suboptimal input parameter for later feed control; in short because the CO_2_ output varies accordingly, to not only fermentation rate, but also the amount of material in the tank. Therefore, CO_2_ does not fully represent the actual fermentation rate at a given time. These limitations do not apply to the RI signal – assuming a well-mixed tank. Having established that the RI signal is proportional to the total sugar concentration in the fermentation broth, any change in the concentration will be reflected by the slope of the RI curve. This insight should make it possible to identify the sugar concentration, at which the fermentation rate – *R*_*ethanol*_*(g/L/h)*, is at its maximum. It follows, at this sugar concentration, that the dRI/dt-value should take the minimum value. This sugar concentration should then be the same as where the CO_2_ emission reaches its maximum value and could thus be applied as a set-point to begin the fed-batch operation. To evaluate the hypothesis a simple batch fermentation on wheat straw hydrolysate was carried out; only stirring, pH and temperature were controlled - Fig. 1. The S-shaped curve represents the total sugar consumption. The outcome of the experiment shows how the peak of the CO_2_ curve, and the steepest point on the RI curve, coincide at 28.5 hours displaying values of 4.5% and 120 g/L respectively. As background noise as well as formed ethanol (and other metabolites) contribute to the RI-signal, the actual sugar concentration is unknown. However, as the CO_2_ output peak coincides with the steepest point of the decline in RI value, the time period for the fastest fermentation rate can be identified. Maintaining this RI value by controlled feeding of fresh media into the reactor, ensures that optimal sugar concentration is maintained for the fermentation regarding fermentation rate. The fermentation finished after roughly 48 hours and displayed a final ethanol yield – calculated as total produced ethanol per total available sugar (*Y*_*SE*_ (g_ethanol_/g_sugar_/0.51 × 100%)) of 90%.

### Establishing a new feed strategy

**Figure 2:**
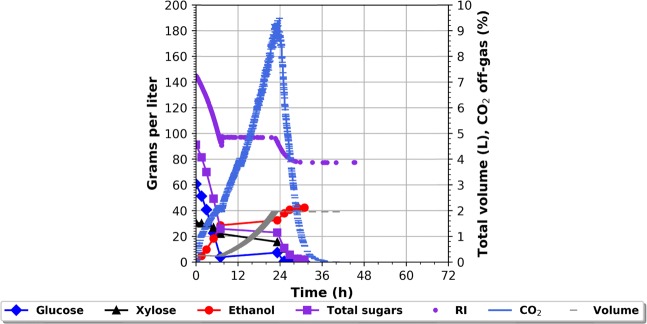
Fermentation profile of a fed batch fermentation on wheat straw hydrolysate (62 g/L glucose, 31 g/L xylose, 5 g/L acetic acid) and corresponding RI data. Fermentation conditions: reaction volume 0.25 L–2 L (1.75 L feed), initial yeast pitch 4 gDW, 3 g/L urea, pH set point 5.2, 32 °C.

Having established that the newly developed RI measuring technique can be used for identifying the sugar concentration, where the fastest fermentation rate takes place (that point is referred to as the RI set point), the next step was to develop and evaluate a feeding strategy that would allow for maintaining that sugar concentration. The online RI-detector was connected to the supervisory control and data acquisition system (BioPAT, MFCS/win) via an open platform communication server, and a feeding protocol was implemented. This protocol turned the feeding off, when the RI was above the RI set point, and gradually increased the feeding as the RI would decrease below the RI set point effectively making the RI value increase above the RI set point. A 0.25 to 2 L fed-batch fermentation on another wheat straw hydrolysate from SEKAB was performed to evaluate the control strategy - Fig. 2. The fermentation conditions were kept at 32 °C, pH 5.2. The cV-110 yeast strain was used with a total pitch of 0.5 g/L i.e. 4 g/L initially in the batch. When the initial batch phase was over after 7 hours, as seen by the local CO_2_ emission peak and slope of the RI curve, the control program automatically detected the decrease in sugar consumption rate i.e. the derivative minimum and the feed pump was started. In order to assure that the derivative minimum indeed had been identified the initiation of feed was halted, allowing the sugar concentration to go below the optimal concentration. Hence, as the feed pump started the sugar concentration would go up and so the RI reading. The RI set point applied was the RI value at that time point i.e. 98 g/L corresponding to 5 g/L glucose + 20 g/l xylose + 30 g/l ethanol (HPLC measurements) and “43 g/L sugar equivalents” background noise. This RI value, and sugar concentration, entailing glucose and xylose co-consumption, was automatically kept throughout the whole fed-batch phase i.e. 7–23 h. When the volume reached 2 L, the feed was depleted, which led to a decrease in the RI signal and CO_2_ emission. This lowering was accompanied by depletion of the residual sugar and increase in ethanol. After about 8 additional hours all the residual sugars in the bioreactor were consumed. The fermentation finished after roughly 32 hours reaching a final *Y*_*SE*_ of 91%. The RI values and corresponding sugar concentrations are shown in Fig. 2. The results show that it is possible to control a fed-batch fermentation applying the online RI monitoring system.

## Applying the new feeding strategy to extend the feed phase beyond the point of a full fermentor – extended fed-batch

**Figure 3:**
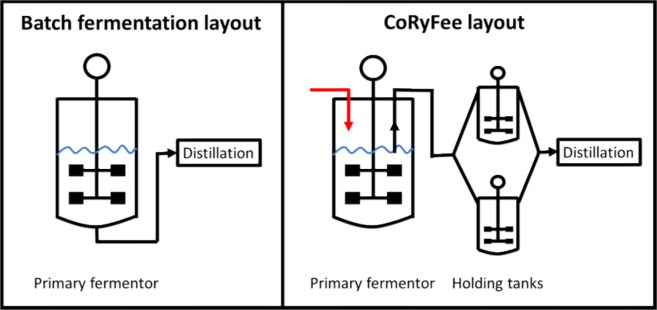
The batch fermentation concept vs. the CoRyFee concept. The primary fermentor is connected to two holding tanks in a parallel manner. The holding tanks allow for stirring and temperature control. A valve makes it possible to switch between the fillings of the holding tanks. All holding tanks are connected to a distillation column.

**Figure 4:**
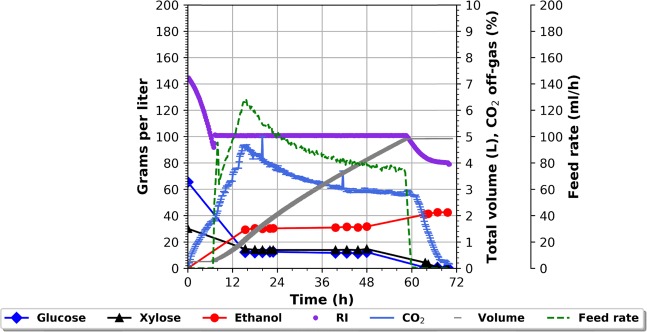
Fermentation profiles of an extended fed-batch fermentation on wheat straw hydrolysate (71 g/L glucose, 40 g/L xylose) and corresponding RI data. Fermentation conditions: reaction volume 0.25 L–5 L (4.75 L feed), working volume of 1 L, initial yeast pitch 0.5 gDW, 3 g/L urea, pH set point 5.5, 32 °C. As the working volume of 1 L is reached at 15 hours in the main fermentor the overflow is directed to the holding tanks.

**Figure 5:**
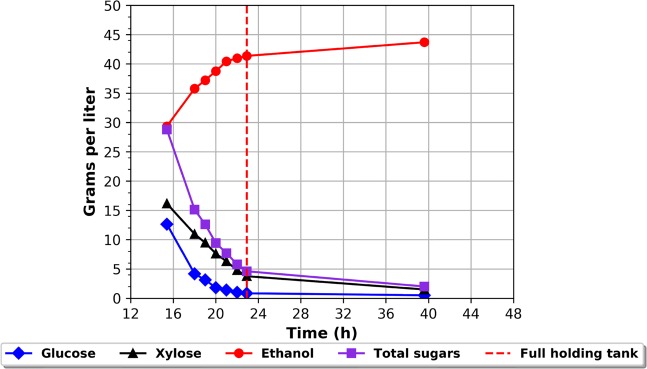
Fermentation profile of one of the holding tanks. The holding tanks were filled up to 0.5 L i.e. 50% of the primary fermentation tank in a water batch at 32 °C and constant stirring. The vertical red dotted line indicates the timepoint at which the bottle was full.

In previous experiments, not shown, the feed- and end-phase of the fermentation showed higher ethanol yields as compared to the batch phase^24,25^. However, upon closer inspection the feed-phase showed the higher ethanol productivity (g_etoh_/gDW/h) compared to the end-phase. It indicates that the feed-phase, from a productivity point of view, should be the longest phase of the entire fermentation. In order to realise the full potential of the new feeding strategy, capable of maintaining the optimal sugar concentration, the fed-batch fermentation setup would need modification. In order to achieve an extension of the feed-phase, three modifications of the fed-batch fermentation setup need to be implemented; 1) the primary fermentor needs to be drained at the same pace as feeding 2) manage the end-phase in order to achieve full fermentation of residual sugars 3) yeast needs to propagate in the same rate as the imposed dilution by drainage. Ad. 1 could be attained by inserting a drain into the primary reactor in such a way that a fixed working volume could be maintained – like a continuous culture. Ad. 2 entails that partly fermented broth, leaving the primary reactor, would be subjected to subsequent secondary fermentation. As the overflow culture still contains significant amounts of sugar, and without the fermentation of these sugars, mainly C5 sugars e.g. xylose, the ethanol yield would be equally reduced. Ad. 3 the substrate subject for fermentation need to contain all necessary yeast growth factors. An appropriate setup for an extended fed-batch, entails a primary fermentor, which allows for drainage into parallel connected holding tanks. In this setup fresh media is fed into the primary fermentor, which also is subjected to pH, temperature and stirring control. The overflow from the primary fermentor is transferred to the holding tank, where the only control is stirring of the culture. The overflow from the main fermentor would thus be directed to the holding tanks in a parallel manner so that once the first holding tank has been filled, the overflow stream from the primary fermentor would be redirected to a second holding tank similar to the first holding tank - Fig. 3. As holding tank two is filled, holding tank one is left to finish the fermentation of the residual sugars left in the overflow broth. As holding tank one has finished, the content will be sent for distillation and the bottle will then be ready for the next filling, which happens when holding tank two is full and needs to be disconnected in order to bring the fermentation to completion. Switching back and forth between the holding tanks should thus enable handling the end-phase of the fermentation. The fermentation conditions were kept at 32 °C, pH 5.5. The cV-110 yeast strain was used with a total pitch of 0.2 g/L i.e. 4 g/L initially in the batch. The initial batch was composed of 250 ml wheat straw hydrolysate supplemented with urea at a concentration of 3 g/L. A feed bottle containing 4.75 L of the same hydrolysate was connected to the main fermentor. Finally, two 0.5 L holding tanks were attached to the main fermentor. The feeding was controlled by the RI signal as previously described. The RI set point was identified and maintained at 100 g /L corresponding to 10 g/L glucose + 15 g/L xylose + 32 g/L ethanol, entailing glucose and xylose co-consumption, measured by HPLC, and ”43 g/L sugar equivalents” background noise - Fig. 4. The figure shows the entire time course of the fermentation in the main fermentor. The events of the batch profile are like the ones described for Fig. 2. Once the main fermentor reached its working volume of 1 L (15 h) the overflow was directed to the 1^st^ holding tank. The filling of the tank lasted 8 hours, at which timepoint the total sugar concentration was 5 g/L (from an initial concentration of 25 g/L). The overflow was then redirected to the 2^nd^ holding tank - Fig. 5. After 60 hours the feed was finished and the end phase fermentation of the main fermentor was initiated. Twelve hours later (72 hours of fermentation) the total sugar concentration decreased to zero while the ethanol concentration increased to 42 g/L. The final RI value reached 80 g/L (sugar equivalents) of background noise. Thus, after 72 hours of fermentation 5 L of hydrolysate had been passed through the main fermentor, i.e. 5 working volumes, and holding tanks with a total yeast pitch of 0.2 g/L and *Y*_*SE*_ of 92%.

## Discussion

In order to make a fair comparison between the different fermentation concepts employed in this study (batch, fed-batch and CoRyFee) a table summarising the fermentation parameters; yeast pitch, ethanol yield, ethanol titres and fermentation rate has been compiled - Table 1. Yeast needed for a fermentation process is one of the more expensive ingredients in terms of the overall costs of the process^19^. The CoRyFee concept reduces the cost of needed yeast by more than 80% as compared to a batch process, and 60% as compared to a fed-batch process. These numbers arrive from the fact that in order to ferment 5 reactor volumes the amount of pitching yeast would be 5 and 2.5 times as much for the batch process and fed-batch process as compared to the CoRyFee based, respectively. This reduction is achieved without any loss in the ethanol yield. On the contrary, the ethanol yield of the processes is 90, 91 and 92% for the batch, fed-batch and CoRyFee, respectively. Also, in terms of productivity the CoRyFee concept shows improvement as compared to the traditional strategies: It has an increased ethanol productivity (*g*_*ethanol*_*/L/h*) compared to batch and fed-batch; 2.5 (if just considering the primary fermentor, and 3.3 if also including the holding tanks) compared to 1.1 and 1.3, respectively. This without including the up/down-time needed in the batch process to empty, clean and prepare the fermentation tank. The fact that the RI measurement includes all the sugars and some of the fermentation products in addition to dissolved salts, is a drawback of the method, however from a control perspective they can easily be subtracted. Thus, from an applied point of view, this is of less importance since it still allows for identifying transition conditions i.e. where the fermentation rate changes.

**Table 1:** Comparison of the different fermentation strategies applied in this study in terms of yeast needed (total yeast pitched/total volume fermented), ethanol yield Y_SE_ (g_ethanol_/g_sugar_/0.51 × 100%), ethanol titres and average volumetric productivity –

	Batch	Fed-Batch	CoRyFee
*Total yeast pitch (g/L)*	1.0	0.5	0.2
*Y*_*SE*_ *(%)*	90	91	92
*Ethanol titre (g/L)*	53.1	42.3	42.6
*R*_*ethanol(*_*(g/L/h)*	1.1	1.3	2.5(3.3*)

asterisk indicates whether the holding tanks are included or not.

In this present work, the CoRyFee based fermentation was sustained for five volumes changes. However, nothing indicate that the continuation of the fermentation was limited by that number of volumes fermented - Fig. 4. On the contrary, the fermentation seemed to close in on a (quasi)steady state. It should, however, be emphasised, that it is not a continuous fermentation. The feed is not constant; hence a more correct term would be extended fed-batch. Also, worth noticing is that during the feed phase there is s glucose and xylose co-consumption – a hallmark of the cV-110 strain. An often-encountered cause to continuous fermentation interruption is contamination, often in the form of lactic acid bacteria^26,27^. However, in the CoRyFee fermentation scheme restarting the fermentation would only be needed if the contamination occurs in the primary fermentation tank. Should the contamination occur in one of the holding tanks, it would be sufficient to address this tank while the rest of the system is left untouched. Interestingly, the RI measurements were unaffected by the presence of suspended particulate matters and changing concentrations of yeast. It is worth noticing that the fermentation seemed to be able to continue indefinitely, however, this also raises the question as to how this is possible with only an added nitrogen source. Traditionally, at least ergosterol and some unsaturated fatty acids would have been expected to be provided, in order to enable the cells to grow anaerobically^28,29^. However, the need for O_2_, in order to fill the need for biosynthesis of ergosterol and unsaturated fatty acids, is very limited. Therefore, if the feed is fully saturated with oxygen it should fulfil the need^30–32^. Another result was that no vitamins were added otherwise known to be necessary for the continuous fermentation of lignocellulose hydrolysate by *S. cerevisiae*^33^. A possible explanation would be the enzymatic hydrolysis of the biomass. The hydrolysing enzymes are often produced by one of the microorganisms *Aspergillus niger*, *Aspergillus oryzae*, *Trichoderma harzianum*, or *Trichoderma reese*^34^. The enzyme broth added often contains plenty of lysed host organism i.e. nutrients for the fermenting yeast. Adding enough of the enzymes to the biomass prior to the hydrolysis could supply the hydrolysate with the necessary components. Nevertheless, it is likely that the fermentation could be optimised with respect to vitamins, trace elements, nitrogen source etc. both with respect to the actual fermentation performance, but also regarding economic considerations^35^. This raises another interesting question, which would be to achieve this optimisation in the cheapest way possible and this would be the natural scope of a future follow-up project.

## Conclusions

This present study demonstrated that it is feasible to monitor and control an extended fed-batch fermentation applying online RI-detection. An optimised C5/C6 fermenting yeast strain, cV-110, was used, displaying C5/C6 co-utilisation and complete sugar depletion together with no xylitol production. Here, a fermentation monitoring technique was developed and applied; based on real-time measured refractive index, an optimal residual sugar concentration could be maintained in the fermentor during the feed phase and a continuous phase. The technique was successfully applied to run a regular fed-batch fermentation. The concept was then expanded, so that the primary fermentor was connected to holding tanks with temperature control and stirring only, such that the overflowing culture broth would be collected allowing the fermentation to complete. This is to the best of the authors knowledge the first time that the refractive index has been applied to monitor and guide a fuel ethanol fermentation. It was possible to run this system stably for more than five fermentor volumes, where we reached a quasi-steady state after four volume changes and an ethanol yield of >90% throughout the run. This was achieved by adding only urea to the hydrolysate and with an initial yeast pitch of less than 0.2 g/L total finished broth (4 gDW/L in the initial batch phase with 25% fermentor filling). The highly efficient fermentation technique has been termed, and patent filed, as the CoRyFee concept. Compared to the standard batch fermentation concept, the CoRyFee concept comprises several additional high value benefits. Apart from the more than 100% increased fermentation rate per fermentor volume, some of the additional benefits are: (**1)** Reduced need of inoculation yeast, (**2)** Reduced need for tank cleaning and sterilisation. Even though the yeast strain cV-110 and wheat straw hydrolysate were used in this case study, nothing, in the opinion of the authors, seem to indicate that the monitoring process should be limited to this specific bioconversion or yeast strain. If measurement is used as input to control a feed, then the technique has widespread application areas; obvious processes where the system could be applied are 2^nd^ ethanol production, as indeed shown here, but with other feed stocks e.g. birch and aspen, otherwise often too hard to ferment. This should be possible as the feed is adjusted to the sugar conversion rate. Also 1^st^ generation ethanol production and yeast propagation could be fields of operation as the concept allows for controlling the sugar concentration. This work thus, offers a new and promising fermentation concept together with an engineered and stable C5/C6 fermenting yeast strain for the ethanol industry.

## Methods

### Yeast strain and maintenance

The cV-110 yeast strain (Terranol A/S, Denmark) was used throughout the studies. It is a strain based on a *S. cerevisiae* Meyen ex E.C. Hansen strain. The organism has been genetically modified by insertion and overexpression of three *S. cerevisiae* genes: *Rki1*, Ribose*-5-phosphate isomerase;* Tkl1 Transketolase and *Tal1 Transaldolase*. An artificially synthesized gene encoding a xylulokinase from *Pichia stipitis* - *Ps-Xks1*, under the control of native *S. cerevisiae* promoter and terminator has been inserted. Further two artificially synthesized genes encoding *LIMR Xylose 1-epimerase* and *LlXI Xylose isomerase* both from *Lactococus lactis* have been inserted. All gene expressions were under the control of native *S. cerevisiae* glycolytic promoters and terminators^9,10^. In addition, the strain has undergone extensive evolutionary engineering in order to optimise its C5/C6 co-fermentation capacity, the tolerance towards inhibitors and eliminating unwanted side products.

All cells in this study were maintained by cultivating them in shake flasks on complex medium containing 10 g/L yeast extract, 20 g/L peptone, 5 g/L MgSO_4_ · 7H_2_O g/L, and 40 g/L ethanol (YPE medium). All raw materials were from Sigma Aldrich unless stated otherwise (Sigma-Aldrich, USA). When stationary phase was reached, 160 g/L sterile glycerol was added, and 1 ml aliquots were stored in sterile vials at −80 °C. These stock cultures were used subsequently as the inoculum for precultures from which all experiments started.

### Hydrolysate

Different batches of wheat straw hydrolysate from SEKAB E-technology (Örnsköldsvik, Sweden) were used in all fermentations. The pre-treatment of wheat straw was performed by SEKAB E-Technology in the Biorefinery Demonstration Plant (BDP), Örnsköldsvik, Sweden. The demonstration unit has a pre-treatment capacity of approx. 1 ton of feedstock (dry weight) per day and is equipped with a continuous steam explosion pre-treatment reactor. Wheat straw was impregnated with sulfuric acid prior to pre-treatment and the subsequent pre-treatment was performed at a temperature, residence time and reactor pH that resulted in a combined severity factor of approx. 1.8. Material was subsequently hydrolysed with a commercially available enzyme preparation after which the solids were separated from liquid phase by means of filtration. The composition of the specific hydrolysate is stated throughout the figure legends. However, average compositions were in the range of 60–90 g/L glucose, 30–40 g/L xylose and 3–6 g/L acetic acid.

### Cell cultivation

A yeast preculture was cultivated overnight on YPD 4%, pH 5.5 glucose media at 30 °C and shaking at 150 rounds per minute. The cells were harvested after all glucose had been depleted as indicated by Glucose MQuant glucose sticks (Merck Millipore, USA). The cells were washed twice in sterile MQ-H_2_O before pitching the fermentor.

### Fermentation control

All fermentations were monitored and controlled by the batch oriented supervisory control and data acquisition system; Sartorius Multi Fermentor Control System MFCS/Win 3.0 (Sartorius Stedim Biotech, Germany).

### Batch cultivation

1 gDW/l cV-110 was pitched in 1 L hydrolysate within a 2 L Biostat B plus laboratory fermentor (Sartorius, Germany). 30 ml/min N_2_ was used as carrier gas through the fermentation broth and merged with 470 ml atmospheric air for dilution of the off gas prior to reaching the gas analyser. Prior to merging, the off gas from the reactor had passed through a condenser with a temperature of 6 °C. Fermentation conditions were kept at 32 °C, stirring rate of 300 rpm, pH was maintained at 5.5 kept with 2 M H_2_SO_4_ and 20% (w/v) NH_3_. Urea was added at a concentration of 3 g/L.

### Fed batch cultivation

cV-110 was cultivated in a 2 L Biostat B plus laboratory fermentor (Sartorius, Germany). 30 ml/min N_2_ was used as carrier gas through the fermentation broth and merged with 470 ml atmospheric air for dilution of the off gas prior to reaching the gas analyser. Prior to merging, the off gas from the reactor had passed through a condenser with a temperature of 6 °C. Fermentation conditions were kept at 32 °C, stirring rate of 300 rpm, pH was maintained at 5.5 kept with 2M H_2_SO_4_ and 10% (w/v) NH_3_. Fresh hydrolysate was fed into the reactor by a Watson SciQ 400 peristaltic pump. The pump was connected to the fermentation tower and controlled by MFCS/Win. Urea was added at a concentration of 3 g/L.

### Extended fed-batch cultivation

The extended fed-batch fermentation was set up like the batch, with extra addition so that when the working volume of the reactor reached a user defined volume, as measured by weight, the overflow would be removed and transferred to a holding tank with temperature control of 32 °C and stirring by a magnetic flee. Urea was added at a concentration of 3 g/L.

### Analyses

Samples for optical density and dry weight were analysed directly while samples for HPLC were kept at −20 °C. Growth was monitored by measuring OD at 600 nm with a Shimadzu UV-1800 spectrophotometer and cell dry weight was determined by extracting a minimum of 5 mL from the culture in a 50 ml falcon tube. The cells were washed three times with MQ-water before the dry matter was determined in a moisture analyser (Mettler Toledo HE73) at a drying temperature of 105 °C. Off gas (CO_2_, Ethanol and O_2_) was measured by an Innova 1316A-3 multi gas monitor that was connected to the fermentation control program (MFCS/Win Sartorius, Germany). The refractive index was continuously measured by a Krüss Process refractometer of the Prb21 series, that had been serially connected to the bioreactor, such that cell broth was passed continuously by the RI detection cell at a rate of roughly 70 cm/second. The RI signal was forwarded to the fermentation control program (MFCS/Win Sartorius, Germany) via an open platform communication server. Glucose, xylose, arabinose, glycerol, and ethanol were separated and quantified by HPLC (Ultimate 3000, Dionex, USA). The compounds were separated with an HPX-87H (Bio-Rad, Hercules, CA, USA) ion exchange column. Separation was performed at 60 °C, with 5 mM H_2_SO_4_ as mobile phase at a flow rate of 0.6 mL/min. A refractive index detector (RID-6A; Shimadzu, Kyoto, Japan) was used for quantification. The calculated carbon recoveries (c-mol/c-mol) were between 97 and 101% for all fermentations.

## Data Availability

Data can be given on request. The IP rights of the cV-110 yeast strain are exclusively owned by Terranol. SEKAB E-technology and Terranol have jointly filed for patent of the fermentation technology, CoRyFee. Hydrolysate can be purchased from SEKAB E-technology upon request.
